# A Framework for Representing, Building and Reusing Novel State-of-the-Art Three-Dimensional Object Detection Models in Point Clouds Targeting Self-Driving Applications

**DOI:** 10.3390/s23146427

**Published:** 2023-07-15

**Authors:** António Linhares Silva, Pedro Oliveira, Dalila Durães, Duarte Fernandes, Rafael Névoa, João Monteiro, Pedro Melo-Pinto, José Machado, Paulo Novais

**Affiliations:** 1 Algoritmi Centre, University of Minho, 4800-058 Guimarães, Portugal; pedro.jose.oliveira@algoritmi.uminho.pt (P.O.); duarte.fernandes@dtx-colab.pt (D.F.); joao.monteiro@dei.uminho.pt (J.M.); pmelo@utad.pt (P.M.-P.); jmac@di.uminho.pt (J.M.); pjon@di.uminho.pt (P.N.); 2 Associação Laboratório Colaborativo em Transformação Digital (DTx Colab), 4800-058 Guimarães, Portugal; 3 Bosch Car Multimédia, 4700-113 Braga, Portugal; rafael.nevoa@pt.bosch.com; 4 Capacity Building and Sustainability of Agri-Food Production, Centro ALGORITMI, University of Trás-os-Montes and Alto Douro, 5000-801 Vila Real, Portugal; 5 Intelligent System Associate Laboratory (LASI), 4800-058 Guimarães, Portugal

**Keywords:** autonomous driving, deep learning methods, LiDAR sensing technology, 3D object detection

## Abstract

The rapid development of deep learning has brought novel methodologies for 3D object detection using LiDAR sensing technology. These improvements in precision and inference speed performances lead to notable high performance and real-time inference, which is especially important for self-driving purposes. However, the developments carried by these approaches overwhelm the research process in this area since new methods, technologies and software versions lead to different project necessities, specifications and requirements. Moreover, the improvements brought by the new methods may be due to improvements in newer versions of deep learning frameworks and not just the novelty and innovation of the model architecture. Thus, it has become crucial to create a framework with the same software versions, specifications and requirements that accommodate all these methodologies and allow for the easy introduction of new methods and models. A framework is proposed that abstracts the implementation, reusing and building of novel methods and models. The main idea is to facilitate the representation of state-of-the-art (SoA) approaches and simultaneously encourage the implementation of new approaches by reusing, improving and innovating modules in the proposed framework, which has the same software specifications to allow for a fair comparison. This makes it possible to determine if the key innovation approach outperforms the current SoA by comparing models in a framework with the same software specifications and requirements.

## 1. Introduction

The field of computer vision has seen significant advancements in recent years, particularly in the area of 3D object detection from point cloud data. However, there is still a need for a general representation framework that can be applied to a wide range of 3D object detection tasks, regardless of the specific sensor or application domain. The development verified in recent years of the computational power offered by cutting-edge GPUs has allowed for the application of deep learning algorithms to detect objects in several domains. One such domain is autonomous driving using light detection and ranging (LiDAR) data, representing a considerable gain in detection efficiency, precision and inference speed [1].

In recent years, there has been significant progress in 3D object detection models based on LIDAR data for self-driving applications. A multitude of frameworks and projects have been proposed, each with its own unique approach to addressing the challenges of detecting and tracking objects in a 3D environment. However, this diversity also poses a challenge when it comes to deploying these models for onboard inference in a self-driving vehicle [2,3].

The misclassification of off-road regions is one of the difficulties with LiDAR-based object detection highlighted in [4]. Finding and classifying off-road areas is essential for safe and accurate autonomous navigation. It also suggests combining high-definition (HD) maps with LiDAR data to overcome this problem. The platform improves the process of item recognition and categorization by adding HD maps, which offer comprehensive information about the road network. The LiDAR system can more easily distinguish between legitimate impediments and off-road areas thanks to HD maps’ comprehensive road geometry data. By enhancing object identification precision and lowering false positives and false negatives, this integration makes autonomous navigation safer and more dependable. Another key idea is that to improve item recognition and classification in the context of automated driving systems, it uses LiDAR’s abilities to capture exact 3D information about the environment and integrate it with HD maps.

One major issue is the enormous variation in software versions, libraries and supported platforms, making it difficult to assemble and deploy these models correctly. Additionally, self-driving requirements must be taken into consideration, such as the need for operationalization with different modules and the limited computational resources available in onboard systems.

Regardless, the 3D object detection models discussed in the literature take point clouds as input and are known to be more complex. These models have a deeper pipeline and process a more significant amount of data. For example, a point cloud usually comprises between 100 k–120 k [3], where each point holds data related to the Euclidean distance and signal reflection, that is, 128 bits to translate each information of each point.

The literature includes recent research such as [3,5,6,7]; it has been suggested that the minimum operating requirements for self-driving applications should include an overall class classification of at least 60 mAP and an inference time of less than 100 ms.

In this context, the need for a standardized and optimized framework for 3D object detection based on LIDAR data becomes even more important. Such a framework could simplify the deployment process, enable better interoperability across different systems and facilitate the development of more efficient and effective self-driving systems.

### Our Contribution

This paper aims to propose a general SoA representation framework for 3D object detection from the point cloud. It supports multiple SoA 3D object detection methods with highly refactored codes for both one-stage and two-stage methods. Also, it enables the implementation and reusing of different approaches with less manual engineering effort by proposing an abstract way of building object detectors. At the same time, it facilitates the implementation of new methods in each module of the framework. By implementing different SoAs, we are trying to facilitate a new approach for the scientific community. In this way, it will be possible to offer a framework for real-time testing inference and measure the trade-off between metrics (mAPvs inference time) in single-framework 3D model objects applied to self-driving applications.

Therefore, the contributions proposed in this paper are as follows:

- An abstract framework for the implementation/representation of edges for 3D object detection models using LIDAR data.
- Less engineer effort to implement new methods in different framework models.
- A simpler way to change hyperparameters and retrain models using YML files.
- An easily represented model using these YML files automatically.

The organization of this paper is as follows: In the next Section 2, some of the state-of-the-art works related to 3D object detection systems and hardware platforms for their implementation are presented. Section 3 shows a four-step method used to select, train and tune a deep learning model for deployment on a hardware device. Section 4 presents the selected 3D object detection model, as well as its deep learning components, specifying the details about the architecture of the target hardware device and the implementation of the hardware components and software. The presentation of performance evaluation results, comparison of results and discussion of these results occur in Section 7. Finally, Section 8 presents the main results achieved in this paper and future work.

## 2. Related Work

In recent years, object detection models in point clouds presented in the literature have been highly improved, and higher and higher detection performance has been achieved. Based on the literature, the most discussed models are divided into two broad categories: approaches based on CNN 3D and approaches based on CNN 2D, where different data representations, backbone networks and multiscale resource learning techniques can be adopted [3].

When it comes to 3D object detection approaches, they can be classified into three types. The first category is based on volumetric representation. The second is based on pillars. Finally, the third is based on raw points. Furthermore, they are novel models recognized by the scientific community that provide innovation in the diverse architecture pipeline, as well as high accuracy and performance in 3D object detection.

The first category, which can be divided into one-stage or two-stage, is usually based on the volumetric representation to discretise the point cloud. The one-stage representation only has a single stage, and SECOND [8] is an example. This 3D convolution-based technique produces item class prediction, bounding box regression and orientation classification. The two-stage representation obtained the same results as the single stage but fine-tuned the bounding box. Examples of two-stage representation are P-RCNN [9], VoxelRCNN [10] and ${\mathrm{PartA}}^{2}$ [11]. Usually, these methods require more resources in terms of computing power because they either use the costly volumetric representation of the point cloud or rely on computationally intensive 3D convolutions.

The second category of models fall under one-stage methods, and use 2D convolutions in place of the computationally intensive 3D convolutions. PointPillars [12] is an example of this approach. To decrease the high computational cost of handling 3D LiDAR data, these models usually compress the data into a 2D projection or organize it into pillars [12]. While these methods are quicker and suitable for real-time applications, they sacrifice detection capabilities by losing some information. This highlights the trade-off between inference time and accuracy.

The third category of methods, such as Point RCNN [13], utilizes a two-stage approach based on raw point data and voxel representation to take advantage of their respective benefits. In the first stage, the network uses voxel representation as input and performs light convolutional operations, which results in a small number of high-quality initial predictions. An attention mechanism effectively combines coordinate and indexed convolutional features of each point in the initial forecast, maintaining both accurate localization and contextual information. The second stage uses the fused feature of interior points to refine the prediction [14].

Accurate object recognition in autonomous vehicles can be considerably improved by utilizing shared visual data from numerous vehicles and infrastructure sensors. This method can get beyond restrictions like occlusion and a narrow field of view by exchanging information with nearby infrastructure and vehicles. Accurate vehicle position, velocity and attitude information are essential to achieve this improvement [4].

The autonomous vehicle kinematics and dynamics synthesis can estimate a vehicle’s side slip angle (SSA), which is an important vehicle state parameter in vehicle dynamics and is based on a consensus Kalman filter. The kinematics and dynamics of the vehicle are very nonlinear; yet, after linearization, the linear system may mimic them well. The vehicle state, which consists of the vehicle’s position, velocity and attitude, is present in the linear system. The first-order differential equation can simulate the vehicle kinematics and dynamics, determining how the vehicle state changes in the linear system. The consensus Kalman filter-based SSA estimation approach can estimate the SSA accurately and robustly. Based on a first-order differential equation, the technique estimates the SSA using the vehicle’s kinematics and dynamics. The nonlinear SSA estimation approach is well approximated by the linear system following linearization. The very accurate SSA estimate approach can be utilized to enhance vehicle control and safety [4]. This method’s accurate estimation of vehicle kinematics, including location, velocity and attitude, can considerably improve autonomous cars’ ability to identify objects. The system may better comprehend the dynamics and behavior of nearby automobiles, pedestrians and other objects in the environment by adding this information into the object detection algorithms. As a result, object detection becomes more accurate, especially in conditions when occlusion and a small field of view present difficulties. So, autonomous vehicles can get over limitations like occlusion and a small field of view by incorporating this information into object detection algorithms. The precision of object identification and the general perception abilities of autonomous vehicles are considerably improved by the precise estimation of vehicle kinematics.

Object detection in vehicle surrounding surroundings or remote sensing provide distinct problems in comparison to natural scene picture detection. Specialized detection techniques are needed in these domains to identify certain things of interest, such as cars, people walking or tassels in UAV footage. The “YOLOv5-Tassel” method is one of the many strategies being investigated by researchers to improve object detection performance in these fields [15]. Improvements including architecture adjustments, data augmentation methods and hyperparameter optimization are included in the YOLOv5-Tassel model. These improvements aim to increase the reliability and precision of tassel detection in UAV photography. The YOLOv5-Tassel model’s performance on a variety of datasets is thoroughly evaluated by the authors, who also compare it to other detection techniques to show how successful it is. The results of the tests show that YOLOv5-Tassel detects tassels in UAV imagery with a high degree of accuracy. When comparing remote sensing with object recognition in natural scenarios, object detection in remote sensing images is more challenging because it calls for the detection of targets from different scenes. Although there are a lot of remote sensing images, there are not as many of them labeled as there are in a dataset of natural scenes, which makes it harder for training models to converge [16].

## 3. Methodology

**Figure 1:**
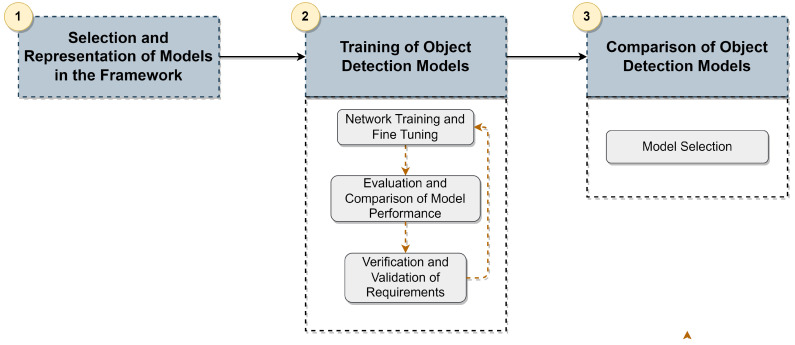
Methodology for object detection model fine-tuning.

To implement/represent the 3D object detection models based on deep learning in the framework, we employed a three-step methodology, which is depicted in Figure 1. (1) Firstly, a set of model architecture and hyperparameter specifications are defined in different configuration files. These files define the specifications of the components of each module in the framework (described in Section 4) as well as the training and test specifications that are then used to build, train and test the object detectors. We chose the models for 3D object detection based on a review of the existing literature, which is outlined in Section 2 and elaborated further in [3]. The framework, described in Section 4, was developed to facilitate the representation of any object detection model.

Once the object detector is built, it is subjected to a training and evaluation pipeline (2), where various optimizations can be performed to enhance the accuracy metrics and fulfill the inference time requirements. In our project, since different components need to operate simultaneously, such as the SLAM algorithm and object detector, we define an overall mAP of 60% and an inference time of less than 100 ms (metrics are always subject to trade-offs). The training and evaluation step can be carried out by changing the training specification in the respective model configuration. The concept behind defining the training and testing parameters in these configuration files is to make it easier to modify them and subsequently submit the object detector to the same training and evaluation pipeline. The pipeline was executed on a server-side node with an Intel Core i9 processor, 64 GB of RAM and a Quadro RTX 8000 GPU. Therefore, the proposed workflow follows an iterative process, where the model is fine-tuned. The training and evaluation steps are repeated whenever necessary until they meet the requirements and satisfy the application requirements. The evaluation and comparison process is carried out using KITTI benchmarks using the validation set on the aforementioned server node. In conclusion, this workflow guarantees that the models meet the application requirements and attain the highest possible accuracy. This procedure identifies a group of potential object detection models for the subsequent step.

After completing step (2) workflow, a comparison phase of the resulting models (step (3)) is conducted to select the model that can ensure a better balance between precision and inference time. The subsequent section presents information on the architecture of the framework, the chosen deep learning models and the parameters used in the fine-tuning process.

## 4. Framework for Representing 3D Object Detection Models

**Figure 2:**
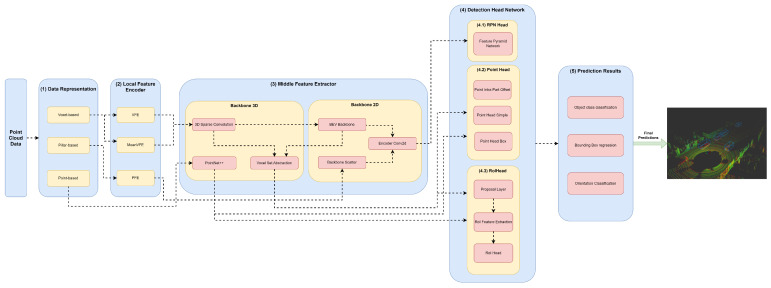
Framework used for the implementation/representation of object detection models.

Our framework’s key innovation is that it facilitates the representation of any object detector through YML configuration files that define their module specifications in each framework component. Moreover, this framework, shown in Figure 2, aims to facilitate the implementation and integration of new modules in each framework component to allow for the comprehensive representation of the different state-of-the-art 3D object detectors.

The first component, (1) data representation, receives the set of points and discretizes them in a set of data structures, such as pillars or voxels, or only passes the set of points to be used by the middle extractor module (3). (2) The local feature encoder receives as input these data structures—more specifically, the set of pillars or voxels—and encodes and concatenates their features. Then, in the middle extractor (3), 3D and/or 2D backbones extract features from local encoded features, which are used by the (4) detection head to predict object class, bounding box offsets and direction (5). (4.1) This detection head based on RPN can be assisted by two modules, a (4.2) point head module and (4.3) region of interest (RoI) head module, which refines the predicted bounding box offsets and orientation. (4.2) The point head module is composed of three networks: a point intrapart offset head [11], a point-based segmentation head for keypoint segmentation [17] and another point-based segmentation head based on [13]. The (4.3) RoiHead module is defined for each state-of-the-art model based on their specificities, but typically it is composed of a proposal layer, which proposes a set of RoIs, a RoI feature extraction that pools the RoI features and a RoI head that predicts RoI class and bounding box offsets.

### 4.1. Point Cloud Data Representation

We receive an unordered set of points $PC=\{p_{1},p_{2},p_{3}\cdots p_{n}\}$, where n>0 and each point *p* is represented as $\left(p^{x},p^{y},p^{z},p^{r}\right)$, where $p^{x},p^{y}$ and $p^{z}$ correspond to coordinates in the three-dimension Cartesian axis and $p^{r}$ is the reflectance value provided by the LiDAR sensor. A point cloud range $PC_{R}$ is a tuple (L,H,W), where *L* consists of $\left(x_{min},x_{max}\right)$, *H* consists of $\left(y_{min},y_{max}\right)$ and *W* consists of $\left(z_{min},z_{max}\right)$. We denote a point cloud subset with respect to $PC_{R}$ as $PC_{R}=\left\{p_{i}:p_{i}\in PC,x_{min}\leq p_{i}^{x}\leq x_{max},y_{min}\leq p_{i}^{y}\leq y_{max},z_{min}\leq p_{i}^{z}\leq z_{max}\right\}$.

#### 4.1.1. Pillar Representation

The framework receives the points in $PC_{R}$ and discretizes them in the X–Y axis, thus creating a set of pillars $PL_{p}=\left\{pl_{1},\;pl_{2},\;pl_{3},\;\cdots ,\;PL_{p}\right\}$, where p=mp, mp is the max number of pillars and $mp\in N^{+}$. Each $PL_{p}$ has a fixed size in $PC_{R}$, and it is represented by a tuple $S_{PL_{p}}=\left(w,h\right)$, where *w* is the width of the pillar along the *x* axis, and *h* is the height of the pillar along the *y* axis. The points are grouped accordingly with the pillar that resides.

To deal with the sparsity problem and save computation, a max number of points per pillar NP is defined. The points are randomly sampled if the number of points in each pillar is higher than NP. On the other hand, zero padding is added in cases of less than NP points.

#### 4.1.2. Voxel-Based Representation

The voxelization process assumes a similar way as proposed in pillar discretization; however, the received points are discretized in the X–Y–Z axis. It allows for the creation of a set of voxels $VL_{j}=\left\{vl_{1},vl_{2},vl_{3}\cdots vl_{j}\right\}$, where j=mv, mv means the max number of voxels and $mv\in N^{+}$, and each $VL_{j}$ assumes a fixed size in $PC_{R}$; and a tuple represents $S_{VL_{j}}=\left(w,h,d\right)$. *w* is the width of the voxel along the *x* axis, *h* is the height of the voxel along the *y* axis and *d* is the depth of the voxel along the *z* axis.

A random sampling strategy is also applied to save computation, and a max number of points per voxel NV is also used. The strategy to sample points or apply zero padding is the same as the pillar representation.

#### 4.1.3. Point-Based

The idea in the point-based strategy is to pass the cropped point cloud, herein denoted as $PC_{R}$, to the middle feature encoder.

### 4.2. Local Feature Encoder

The local feature encoder receives the data representation structures DS, such as pillars, denoted as PL; voxels, VL; or just the set of points of cropped area, $PC_{R}$. Then, a set of methods are applied to obtain features and produce dense tensors in the case of pillar feature network (PFN) and voxel feature encoder (VFE) or calculate these features by simply calculating the mean values of point coordinates within each voxel using the mean VFE method.

#### 4.2.1. Pillar Feature Network

The features of each pillar, PL, are augmented in a tensor $D=(x,y,z,r,x_{c},y_{c},z_{c},x_{plc},$

$y_{plc})$, where *c* describes the distance to the arithmetic mean of all points in PL, and plc is the offset distance from the $Pl_{x,y}$ center.

For this purpose, (1) the pillar feature network (PFN) receives the pillar augmented features as input and applies linear transformations to each point, herein described as $linear\left(Pl_{in}\right)=Pl_{out}$, where $Pl_{in}$ corresponds to the initial tensor $Pl_{in}=\left(P,N,D_{in}\right)$, and $Pl_{out}$ to the output tensor. In $Pl_{out}$, all but the last dimension are the same shape as the input. Dimension $D_{out}$ results from the linear transformation of $D_{in}$, thus producing $Pl_{in}=\left(P,N,D_{out}\right)$. Then, batch-norm and ReLU are applied to this tensor. Afterwards, all resulting features are aggregated. This process allows for the generation of a dense tensor to represent the pillar as a tuple (D,P,N), where *D* is the above-mentioned augmented point, *P* is the number of non-empty pillars per batch and *N* is the number of points per pillar. Next, max pooling operations over the channels are used to generate a tensor of size $\left(D_{out},P\right)$.

#### 4.2.2. Voxel Feature Encoder

Similar to PFN, the points in each voxel, $VL_{j}=\left\{pt_{i}=\left(x_{i},y_{i},z_{i},r_{i}\right)\in R^{4}\right\},i=\left\{1,2,\cdots ,NV\right\}$, are augmented by calculating offset distance of the point to the $VL_{x,y,z}$ center, herein denoted as vlc, which generates the tensor $VL_{j}=\left\{pt_{i}=\left(x_{i},y_{i},z_{i},r_{i},x_{vlc},y_{vlc},z_{vlc}\right)\in R^{7}\right\},i=\left\{1,2,\cdots ,NV\right\}$, where NV as mentioned before is the max number of points per Voxel. Afterwards, each $pt_{i}$ is subject to VFE layers $VFEL_{l}$, where l≥1. Each $VFEL_{l}$ is composed by a set of transformations, where linear transformation, batch-norm and ReLU are applied. Then, all points features of $VL_{j}$ resulting from the above-mentioned transformations, herein described as $pf_{j}$, are aggregated. Each $pf_{j}$ can be described as $pf_{j}\in R^{out}$, where out is the output dimension that results from the linear transformation of all points $pt_{i}$. The output size out resulting from the linear transformation can be described as $out_{l}=F_{o}/2$, where $F_{o}=\left\{f_{1},f_{2},\cdots ,f_{o}\right\},F_{o}\in N^{+}$ means the output features of a specific VFEL index *l*. Then, all point features $PF,pf_{j}\in PF$ are subject to a max pooling operation over the channels. The output tensor is described as $pfr_{m}\in R^{out}$, where m=1. Afterwards, a repeat process of the above tensor is performed repeat(pfr,k) in $VL_{j}$, which means the repeat point feature resulted from max pooling *k* times, where k={1,2,3,⋯,NV}. Each $pfr_{k}$ is augmented with $pf_{j}$ to generate $pfo_{j}=\left(pfr_{k},pf_{j}\right)\in R^{2out},k=\left\{1,2,\cdots ,NV\right\}$ and j={1,2,⋯,NV}. The set of features for each voxel can be described by the tuple $VL_{out}=\left\{VL_{j}=stack\left(pfo_{j}\right)\right\}$, where j={1,2,3,⋯,NV}, $stack=(pfo_{1}\times pfo_{2}\times \cdots pfo_{j})$, and applies linear, batch-norm, ReLU and max pooling to each $VL_{j}$. Thus, $VL_{j}\in R^{F}$ means that $VL_{j}$ has the output dimension *F*, the output feature of the last VFE layer.

Finally, it generates a list of obtained voxel features $VLA_{out}$, $VLA_{out}=\{VL_{j}=\left\{vl_{1},\;vl_{2},\;\cdots ,\;vl_{j}\right\},VL_{j}\in R^{F},F=f_{o}$, where $VLA_{j}$ is the above-mentioned augmented features of all voxels.

#### 4.2.3. Mean Voxel Feature Encoder

Mean VFE receives a set of voxels VL, sums all points residing in each voxel in a specific axis and divides by the number of points of each one. This operation can be described as $VLM_{out}≜\{mean{\left(vl_{j}\right)}_{k=0}^{mv}=\{ptf_{i}=(\sum {\left(ptx\right)}_{i=1}^{nv}|count\left(ptx\right)$, $\sum {\left(pty_{i}\right)}_{i=1}^{nv}|count\left(pty\right)\;,\sum {\left(ptz_{i}\right)}_{i=1}^{nv}|count\left(ptz\right)$, $\sum {\left(ptr_{i}\right)}_{i=1}^{nv}|count\left(ptr\right))\in R^{4}\}\},k=[0,NV[,count\left(ptx\right)\to ptx\in vl_{j},$ $count\left(pty\right)\to pty\in vl_{j},count\left(ptz\right)\to ptz\in vl_{j},count\left(ptr\right)\to ptr\in vl_{j}$. nv corresponds to the total number of points of the voxel $vl_{j}\in VL$ in a given axis; mv the max number of voxels; and $ptf_{i}\in VLPF$ corresponds to a resulting point. This strategy considers the voxel-wise features of a new voxel center $VL_{x,y,z,r}$ and approximate equivalence to raw point cloud data. The idea herein is to process the voxel-wise features in the middle feature encoder more efficiently, especially by the 3D sparse convolutions, since they generate mv (max number of voxels, as described in Section 4.1) number of non-empty voxels.

### 4.3. Middle Feature Extractor

The (3) middle feature extractor is responsible for extracting more features from the (2) local feature encoders to provide more context for the shape description of objects for the networks of the detection head module. Various methods are used; herein, we separated them into 3D backbones and 2D backbones, which will be described in more detail below.

#### 4.3.1. Backbone 3D

A variety of methods resort to 3D backbones using the sparse CNN component. Also, models can use a voxel set abstraction 3D backbone, aiming to encode the multiscale semantic features obtained by sparse CNN to keypoints. Others use PointNet++ with multiscale grouping for feature extraction and to obtain more context to the shape of objects, and then pass these features to the (4) detection head module.

**3D Sparse Convolution**

The 3D sparse convolution method receives the voxel-wise features of VFE, $VLA_{out}$ or mean VFE, $VLM_{out}$.

This backbone is represented as a set of blocks BLC, in the form $\left\{blc_{1},blc_{2},\ldots blc_{m}\right\}$, where m=6. Each block $blc_{j}\in BLC,j=m$ can be defined by a set of sparse sequential operations denoted as $SSQ_{s}=\left\{ssq_{1},\;ssq_{2},\;ssq_{3},\;\cdots ssq_{s}\right\},s\geq 1$. Each $SSQ_{s}$ is described by ((SuM→¬SpC)∨(SpC→¬SuM)),Bn,RL), where SuM means submanifold sparse convolution 3D [18], SpC means spatially sparse convolution 3D [19], Bn means 1D batch normalization operation and RL represents the ReLU method. The last method assumes the standard procedure, as mentioned in [20].

In our framework, the set of blocks assumes the following configurations:

- The input block $blc_{1}$ can be described by $blc_{1}=\left\{sq_{1}=\left(SuM,\;BN,\;RL\right)\right\}$;
- The next block is represented in the form $blc_{2}=\left\{sq_{1}=\left(SuM,\;BN,\;RL\right)\right\}$;
- The block 3 is represented as $blc_{3}=\{sq_{1}=\left(SpC,\;BN,\;RL\right),\;sq_{2}=\left(SuM,\;BN,\;RL\right)$, $sq_{3}=\left(SuM,\;BN,\;RL\right)\}$;
- The block 4 is denoted as $blc_{4}=\{sq_{1}=\left(SpC,\;BN,\;RL\right),\;sq_{2}=\left(SuM,\;BN,\;RL\right)$, $sq_{3}=\left(SuM,\;BN,\;RL\right)\}$;
- The block 5 is denoted as $blc_{4}=\{sq_{1}=\left(SpC,\;BN,\;RL\right),\;sq_{2}=\left(SuM,\;BN,\;RL\right)$, $sq_{3}=\left(SuM,\;BN,\;RL\right)\}$;
- The last block is defined by $blc_{6}=\left\{sq_{1}=\left(SpC,\;BN,\;RL\right)\right\}$.

The batch normalization Bn element is defined by (InB,ep,mn), which represents the formula in [21]. InB represents the input features, which are the output features of submanifold sparse or spatially sparse convolutions 3D, so that (OutS→¬OutM∨OutM→¬OutS). ep represents the eps, and mn the momentum values. These values are defined in Table 1.

The element SpC can be represented as (InS,OutS,KsS,StS,PdS,DlS,OpS). InS represents the input features of SpC, and it is denoted as SpC∈N+,InS=OutM, where OutM represents the output features of submanifold sparse Conv3D. The element OutS represents the output features resulting from applying SpC. KsS means kernel size of a spatially sparse convolution 3D, and it is denoted as $KsS_{s}=\left\{KsS_{1},\;KsS_{2},\;KsS_{s}\right\}$, where s={1,⋯,3}, $KsS_{s}\in N+$ and $ksS_{s}=ksS_{s+1}$. The stride StS can be described as a set $StS_{r}=\left\{sts_{1},\;sts_{2},\;sts_{r}\right\},r=\left\{1,\;\cdots ,\;3\right\},StS_{r}\in N+$ and $sts_{r}=sts_{r+1}$. PdS designates padding, and a set can define it $PdS_{v}=\left\{pds_{1},\;pds_{2},\;pds_{v}\right\},v=\left\{1,\;\cdots ,\;3\right\},Pd_{v}\in N+$, $pds_{v}=pds_{v+1}$. DlS means dilation, and can be defined as a set $DlS_{l}=\left\{dls_{1},\;dls_{2},\;dls_{l}\right\}$, $l=\left\{1,\;\cdots ,\;3\right\},DlS_{l}\in N+,dls_{l}=dls_{l+1}$. The output padding OpS is represented as a in the form $OpS_{a}=\left\{ops_{1},\;ops_{2},\;ops_{a}\right\},a=\left\{1,\;\cdots ,\;3\right\},OpS_{a}\in N+$ and $ops_{a}=op_{a+1}$. The configurations used in our framework are represented in Table 2.

SuM is represented by (InM,OutM,ksM,StM,PdM,DlM,OpM) [18]. InM represents the input features passed by (2) the local feature encoder or by the last sparse sequential block $Sq_{s}$, and OutM represents the output features of SuM. Thus, InM∈N+,InM=4 in the case of the local encoder being mean VFE, otherwise In=F, where *F* represents the output features of the VFE network. Also, InS can be represented by InM=OutM and InM=OutS, where OutS represents the output features of a SpC. The element Ks represents the kernel size, which can be defined as $Ks_{t}=\left\{ks_{1},\;ks_{2},\;ks_{t}\right\}$, where t={1,⋯,3}, $Ks_{t}\in N+$ and $ks_{t}=ks_{t+1}$. StM means stride, and can be defined as a set $St_{r}=\left\{st_{1},\;st_{2},\;st_{r}\right\},r=\left\{1,\;\cdots ,\;3\right\}$, $St_{r}\in N+$ and $st_{r}=st_{r+1}$. PdM represents padding, and a set can describe it in the form $Pd_{p}=\left\{pd_{1},\;pd_{2},\;pd_{p}\right\},p=\left\{1,\;\cdots ,\;3\right\},Pd_{p}\in N+,pd_{p}=pd_{p+1}$. Dl means dilation, and can be described as a set $Dl_{d}=\left\{dl_{1},\;dl_{2},\;dl_{d}\right\},d=\left\{1,\;\cdots ,\;3\right\},Dl_{d}\in N+,dl_{d}=dl_{d+1}$. Op represents the output padding, and a set describes it in the form $Op_{u}=\left\{op_{1},\;op_{2},\;op_{p}\right\}$, $u=\left\{1,\;\cdots ,\;3\right\},Op_{u}\in N+$ and $op_{u}=op_{u+1}$. The configurations used in our framework are represented in Table 3.

The hyperparameters used in each $blc_{j}$ are defined in Table 7.

Finally, the output spatial features SP are defined by SP∈R, where SP is defined by a tuple (B, C, D, H, W). *B* represents the batch size; *C* the output features of $blc_{5}$ represented in SpC as OutS; *D* depth; *H* height; and *W* width.

**PointNet++**

We use a modified version of PointNet++ [9] based on [13] to learn undiscretized raw point cloud data (herein denoted as $PC_{R}$) features in multiscale grouping fashion. The objective is to learn to segment the foreground points and contextual information about them. For this purpose, a set abstraction module, herein denoted as $SA_{M}$, is used to subsample points at a continuing increase rate, and a feature proposal module, described as $FP_{M}$, is used to capture feature maps per point with the objective of point segmentation and proposal generation. A $SA_{M}$ is composed of $SA_{M}=\left\{ptn_{1},ptn_{2},\;\cdots ,\;ptn_{g}\right\},g\in N^{+}$, g={1,2,⋯,4}, where ptn means PointNet set abstraction module operations. Each $ptn_{g}\in PTN$ is represented by (QGL,ML), where QGL corresponds to query and grouping operations to learn multiscale patterns from points, and ML is the set of specifications of the PointNet before the global pooling for each scale.

QGL means ball query operation QL followed by a grouping operation GL. It can be defined by the set $\left\{qgl_{1},\;qgl_{2}\right\}$, where $qgl_{1}$ and $qgl_{2}$ correspond to two query and group operations. A ball query QL is represented as (R,NS,P,CP), where *R* means the radius within all points will be searched from the query point with an upper limit $NS,NS\in N^{+}$, in a process called ball query; *P* means the coordinates of the point features in the form $PF=\{pf_{n}=\left(x_{n},\;y_{n},\;z_{n},\;\right)\in R^{3}\},n\in N$ that are used to gather the point features; CP represents the coordinates of the centers of the ball query in the form $CP=\left\{cp_{p}=\left(xc_{p},yc_{p},zc_{p}\right)\in R^{3}\right\},p\in N^{+},p\leq n,p=\left\{1,\;2,\;\cdots \;,4\right\}$, where xc, yc, and zc are center coordinates of a ball query. Thus, this ball query algorithm searches for point features *P* in a radius *R* with an upper limit of NS query points from the centroids (or ball query centers) CP. This operation generates a list of indices ID in the form $\left\{id_{1},\;id_{2},\;\cdots ,\;id_{x}\right\}$, $x\geq 1,id_{x}\in ID,id_{x}\in N^{NCP\times NS}$, where NCP corresponds to the number of CP. ID represents the indices of point features that form the query balls. Then, a grouping operation GL is performed to group point features, and can be described by (PF,ID), in which PF and ID correspond to point features and indices of the features to group with, respectively. In each QGL of a ptn, the number of centroids NCP will decrease, so that $NCP_{p}>NCP_{p+1},p=\left\{1,\;2,\;\cdots ,\;4\right\},NCP\in N^{+}$, and due to the relation of the centroids in ball query search, the number of indices NID and corresponding point features will also decrease. Thus, in each ptn, the number of points features is defined by $NP_{n}>NP_{n+1},NP_{n+1}=NCP_{p},p>1$. The number of centroids defined in QGL during ptn operations is defined in Table 4.

Afterwards, an ML is performed, defined by a set of specifications of the PointNet before the QGL operations. The idea herein is to capture point-to-point relations of the point features in each CP local region. The point feature coordinate translation to the local region relative to the centroid point is performed by the operation $LR=\left\{fr_{f}=\left(px_{f}-xc_{f},\;py_{f}-yc_{f},\;pz_{f}-zc_{f}\right)\in R^{3}\right\},f=\left\{1,\;2,\;\cdots ,\;NS\right\}$. px, py and pz are coordinates of point features PF as mentioned before, and xc, yc and zc are coordinates of the centroid center. ML can be defined by a set $SQ=\{sq_{1},sq_{2}\}$ that represents two sequential methods. Each SQ is represented by the set of operations $OP=\{op_{s}=\left(C2D\;,Bn2D,\;RL\right)\}$, s={0,1,⋯,3}, where C2D means convolution 2D, Bn2D 2D batch normalization and RL represents the ReLU method. C2D is defined by (InC2D,OutC2D,KsC2D,SC2D). InC2D, where $InC2D\in N^{+}$ represents the input features that can be received by QGL or by the output features $OutC2D,OutC2D\in N^{+}$ of the $op_{s-1}$, KsC2D the kernel size, and SC2D represents the stride of the convolution 2D. The kernel size KsC2D is defined by the set $\left\{ksc2d_{1},ksc2d_{2}\right\},ksc2d_{1}=ksc2d_{2}$ and $\forall op_{s}\in SQ,SQ\in ML,ML\in PTN,ksc2d_{1}=1$. Also, the stride SC2D is represented by a set $\left\{sc2d_{1},sc2d_{2}\right\},sc2d_{1}=sc2d_{2}$ and $sc2d_{1}=1,$ with $\forall op_{s}\in SQ,SQ\in ML,ML\in PTN$. The set of specifications used in our models regarding OP are summarized in Table 5. $ptn_{i}\in PTN$ can be defined as:

(1) $$\begin{array}{c} PTN≜\{ptn_{i}=max\left(ML\left(SG\left(pf_{i}\right)\right)\right)\}, \end{array}$$

where max denotes max pooling, SG denotes random sampling of $pf_{i}$ features and ML denotes the multilayer perceptron network to encode features and relative locations.

Finally, a feature proposal $FP_{M}$ is applied employing a set of feature proposal modules $\left\{fp_{1},\;fp_{2},\;\cdots ,\;fp_{m}\right\},m=\left\{1,\;2,\;\cdots ,\;4\right\},m\in N^{+}$. Each $fp_{m}\in FP_{M}$ is defined by the element SQ as defined above. Also, the element SQ assumes a set $\left\{sq_{1},sq_{2}\right\}$, and each SQ has the same operations, with the only difference in the element *s* that describes the number of operations, assuming s={1,2} instead of s={1,2,3}. The configurations used in our models are summarized in Table 6.

**Voxel Set Abstraction**

This method aims to generate a set of keypoints from given point cloud $PC_{R}$ and use a keypoint sampling strategy based on farthest point sampling. This method generates a small number of keypoints that can be represented by $K≜\{p_{j}=\left(x_{j},y_{j},z_{j}\right)\in R^{B*3}\}$, j=[1,NK], where NK is the number of points features that have the largest minimum distance, and *B* the batch size. The farthest point sampling method is defined according to a given subset $PA≜\left\{pa_{j}=\left(xa_{j},ya_{j},za_{j}\right)\right\},j=\left\{1,2,\cdots ,M\right\},PA\subset PF$, where *M* is the maximum number of features to sample, and subset $PB≜\{pb_{k}\left(xb_{k},yb_{k},zb_{k}\right)\}$, k={0,1,2,⋯,N},PB⊆PF, where *N* is the total number of points features of PF; the point distance metric is calculated based on $D≜\{d_{i}=\left\{{\left(xb_{k}-xa_{j}\right)}^{2}+{\left(yb_{k}-ya_{j}\right)}^{2}+{\left(zb_{k}-za_{j}\right)}^{2}\right)\}\},i\leq M$. Based on *D*, an operation $SM≜\{sm_{k}=\left\{min\left(d_{i},sm_{i-1}\right)\right\}\},k\leq M,i\leq N$ is performed, which calculates the smallest value distance between $d_{i}$ and $sm_{i-1}$. $sm_{k}\in SM$, k<N and SM represent the list of the last known largest minimum distances of point features. Assuming $sm_{k}=sm_{i-1}|d_{i}<sm_{i-1}$, it returns the index $IDX=\{idx_{k}=\left(i-1\right)\},$. Based on $sm_{k}=\left\{d_{i}|d_{i}>sm_{i-1}\right\}$, thus $IDX=\{idx_{k}=\left(i\right)\}$. Finally, this operation generates a set of indexes in the form $IDX≜\left\{idx_{0},idx_{1},\cdots ,idx_{m}\right\},idx_{m}\in IDX,m\leq M$, and $IDX\in R^{B*M}$, where *B* corresponds to the batch size and *M* represents the maximum number of features to sample. The keypoints *K* are given by $K≜\{pf_{idx_{0}},\;pf_{idx_{1}},\;\cdots ,\;pf_{idx_{m}}\}$.

These keypoints *K* are subject to an interpolation process utilizing the semantic features encoded by the 3D sparse convolution as SP. In this interpolation process, these semantic features are mapped with the keypoints to the voxel features VL that reside. Firstly, this process defines the local relative coordinates of keypoints with voxels VL by means $VLI≜\left\{vli_{i}=\left(\frac{\left(kx_{i}-PC_{Rx_{min}}\right)}{vlx_{k}},\;\frac{\left(ky_{i}-PC_{Ry_{min}}\right)}{vly_{k}}\right)\in R^{2}\right\},\;k=[0,\mathrm{NK}[,\;i=[0,\mathrm{NV}[$. Then, a bilinear interpolation is carried out to map the point features SP from 3D sparse convolution in a radius *R* with the VLB, the local relative coordinates of keypoints. This is perform $\mathrm{PR}≜\left\{\forall \mathrm{sp}\leq R,sp\in \mathrm{SP}|R=\left(\mathrm{xr},\mathrm{yr}\right)\in R^{2},\;{\mathrm{sp}}_{i}=\left({\mathrm{pfx}}_{i},{\mathrm{pfy}}_{i}\right)\right\},\;i=[0,\;\mathrm{NK}[$. Afterwards, indexes of points are defined according to $vlia\in VLI|vlia_{i}={\mathrm{vli}}_{i}$ in the form (xa,ya) and another vlib≜(xb=(xa+1),yb=(ya+1)). The expression that gives the features $sp_{i}$ from the BEV perspective based on vlia and vlib is the following:

- $SBEVA≜(sp_{vliax},sp_{vliay})$
- $SBEVB≜(sp_{vlibx},sp_{vliay})$
- $SBEVC≜(sp_{vliax},sp_{vliby})$
- $SBEVD≜(sp_{vlibx},sp_{vliby})$

Thus, the weights between these indexes $vlia_{i}$, $vlib_{i}$ and $vli_{i}$ are calculated, as follows:

- $WA≜\{\left(vlix_{i}-prx_{i}\right)\times \left(vliy_{i}-vliy_{i}\right)\}$;
- $WB≜\{\left(vlix_{i}-prx_{i}\right)\times \left(vliy_{i}-vliya_{i}\right))\}$
- $WC≜\{\left(vlix_{i}-prax_{i}\right)\times \left(vliby_{i}-vliy_{i}\right))\}$
- $WD≜\{\left(vlix_{i}-prax_{i}\right)\times \left(vliy_{i}-vliay_{i}\right))\}$

Finally, the bilinear expression that gives the features $sp_{i}$ from the BEV perspective is $PFBEV≜\left(sbeva_{i}*wa_{i}\right)+\left(sbevb_{i}*wb_{i}\right)+\left(sbevc_{i}*wc_{i}\right)+\left(sbevd_{i}*wd_{i}\right)$, where $sbeva_{i}\in SBEVA$, $sbevb_{i}\in SBEVB$, $sbevc_{i}\in SBEVC$, $sbevd_{i}\in SBEVD$. Also, $wa_{i}\in WA$, $wb_{i}\in WB$, $wc_{i}\in WC$, $wd_{i}\in WD$, and i=[0,NV[.

The local features of $pfbev_{j}\in PFBEV$ are indicated by $vlb_{i}=|vl_{k}-sp_{i}|,\;k=[0,\mathrm{NK}[$, i=[0,NV[ and aggregated using PointNet++ according with their specification defined above. They will generate PTN, which are voxel-wise features within the neighboring voxel set $vli_{i}$ of $sp_{i}$, transforming using PointNet++ specifications. This generates $ptn_{i}\in PTN$ according to $PTN≜ptn_{i}=ptn_{0},\cdot ,ptn_{NK}$, and each $ptn_{i}$ is an aggregate feature of 3D sparse convolution $sp_{i}$ with $pfb_{i}$ from different levels according to Table 4.

#### 4.3.2. Backbone 2D

Two-dimensional backbones are used to extract features from 2D feature maps resulting from a PFN component, such as those used by PointPillars, and to readjust the objects back to LiDAR’s Cartesian 3D system with minimal information loss utilizing a backbone scatter component. Also, models can compress the feature map of 3D backbones into a bird’s-eye view (BEV) feature map employing a BEV backbone and use an encoder Conv2D to perform feature encoding and concatenation. Such methodology is employed by models such as by SECOND, PV-RCNN, ${\mathrm{PartA}}^{2}$ and Voxel-RCNN.

**Backbone Scatter**

The features resulting from the PFN are used by the PointPillars scatter component, which scatters them back to a 2D pseudoimage of size $\left(D_{out},H,W\right)$, where *H* and *W* denote height and width, respectively.

**BEV Backbone**

The BEV backbone module receives 3D feature maps from 3D sparse convolution and reshapes them to the BEV feature map. Admitting the given sparse features SP≜(B,C,D,H,W), the new sparse features are (B,C×D,H,W). The BEV backbone is represented as a set of blocks BLC, in the form $blc_{1},blc_{2},\ldots blc_{m}$, where m≥1. Each block $blc_{j}\in BLC,j\leq m$, is represented by (n,F,U,S). The element *n* represents the number of convolutional layers in $BLC_{j}$. The set of convolutional layers *C* in $BLC_{j}$ is described as a set $\left\{c_{1},c_{2},c_{3}\ldots c_{n}\right\}$, where n≥1. *F* represents the number of filters of each $c_{i}\in C,i\leq n$, *U* is the number of upsample filters of $c_{i}$. Each of the upsample filters has the same characteristics, and their outputs are combined through concatenation. *S* denotes the stride in $c_{1}$. If S>1, we have a downsampled convolutional layer ($c_{1}$). There are a certain convolutional layers ($c_{i}$, such that i>1) that follow this layer. batch-norm and ReLU layers are applied after each convolutional layer.

The input for this set of blocks BLC is spatial features extracted by 3D sparse convolution or voxel set abstraction modules and reshaped to the BEV feature map.

**Encoder Conv2D**

Based on features extracted in each block $blc_{j}$ and after upsampling based on U=2D, where *D* means the downsample factor of the convolution layer *C*, the upsample features $u_{j}\in U$, j=[0,m[ are concatenated, such that $UF≜\mathrm{cat}(u_{j})$, where cat means $u_{j}+u_{j+1},j=[0,\;m[$.

### 4.4. Detection Head

After that, the (4) detection head component receives the 2D encoded features as input and performs operations based on three modules: RPN head, point head, and RoI head.

#### 4.4.1. RPN Head

Based on the 2D encoded features, a set of convolutions to predict class labels, regression offsets and direction are performed. Thus, a set of 1 × 1 convolutions $C1x=\{c1x_{1},\;c1x_{2},\;\cdots ,\;c1x_{k}\}$, where k=3, is applied. Each $c1x_{k}$ can be represented by C2D≜(IC,OC,KS), where C2D means convolution 2D, IC input channels, OC output channels and KS kernel size. $c1x_{1}$ is the class prediction convolution, and can be described by (UF,NA×NC,1), where NA means number of anchors per location and NC number of target classes to predict. $c1x_{2}$ is the convolution for bounding box offset regression, and can be defined by (UF,NA×NC×7,KS), where it generates two anchors NA for each class NC and seven bounding box offsets. Finally, $c1x_{3}$ is performed based on (UF,NA×NB,KS) where NA represents the same number of anchors per location, as previously mentioned; NB represents the number of bins per anchor location; and KS represents kernel size.

The figure representing our baseline network for each block can be seen in Figure 2. We use three blocks with a BEV backbone for PointPillars, while for the other models, we use two blocks. Each block is represented as described in Table 7. Table 8 describes the configuration of the RPN head.

**Table 7:** The different block configuration ($blc_{j}\in BLC$) used. N.A.—not applicable.

Models	${\mathrm{blc}}_{1}$	${\mathrm{blc}}_{2}$	${\mathrm{blc}}_{3}$
PointPillars	(3, 64, 128, 2)	(5, 128, 128, 2)	(5, 128, 128, 2)
SECOND	(5, 64, 128, 1)	(5, 128, 256, 2)	N.A.
PV-RCNN	(5, 64, 128, 1)	(5, 128, 256, 2)	N.A.
PointRCNN	N.A.	N.A.	N.A.
PartA^2^	(5, 128, 256, 2)	(5, 128, 256, 2)	N.A.
VoxelRCNN	(5, 128, 256, 2)	(5, 128, 256, 2)	N.A.

#### 4.4.2. Point Head

Different implementations of point head have been proposed to refine RPN predictions or generate class labels, bounding box regression offsets and direction. It can be composed of a class layer regression CR in the form CR≜linear(IN,OT) and bounding box layer BBR described as PR≜linear(IN,OT). The point class layer CR provides the segmentation score of foreground points, and PR gives the relative location of foreground points as $PR≜\{pr_{p}=\left(x_{f},y_{f},z_{f}\right)\}$ and calculated based on a foreground point $fp_{p}=\left(x_{p},y_{p},z_{p}\right)$ using $\left\{\left(x_{t}=\frac{\left(x_{p}-x_{c}\right)}{w}+0.5,\;\frac{y_{t}=(y_{p}-x_{c})}{l}+0.5,\;z_{t}=\left\{\frac{\left(z_{p}-z_{c}\right)}{h}+0.5\right\},\left(cos{\left(\theta \right)}_{p}-cos{\left(\theta \right)}_{c},sin{\left(\theta \right)}_{p}-sin{\left(\theta \right)}_{c}\right)\right)\right\}$, where $x_{c}$, $y_{c}$, $z_{c}$ are center coordinates of the bounding box; *h*, *w*, and *l* means height, width and length of the bounding box, respectively; and θ is the box orientation in bird view.

Firstly, bounding box targets are normalized in a canonical coordinate system by first checking if the given points $PT≜p_{i}=\left(x_{i},y_{i},z_{i}\right),PT\in bb_{k}$ are within the bounding box $bb_{k}≜\left(xc_{i},yc_{i},zc_{i},dx_{i},dy_{i},dz_{i},\theta_{i}\right)$ by performing $((\frac{|x_{i}-xc_{k}|}{2}+0.00001||x_{i}-xc_{k}|<dx_{i}\;\&\frac{|y_{i}-yc_{k}|}{2}+0.00001\left||y_{i}-yc_{k}|<dy_{i}\right),$ where if the given statement is true, the local $lxn_{i}$ and $lyn_{i}$ are calculated. The operation is $lxn_{i}=\left(\left(x_{i}-xc_{k}\right)\times \left(cos\left(-\theta_{i}\right)\right)\right)+\left(\left(y_{i}-yc_{k}\right)\times \left(-sin\left(-\theta_{i}\right)\right)\right)$ and $lny_{i}=\left(\left(x_{i}-xc_{k}\right)\times \left(sin\left(-\theta_{i}\right)\right)\right)+\left(\left(y_{i}-yc_{k}\right)\times \left(cos\left(-\theta_{i}\right)\right)\right)$. Then, we determine the local relative coordinate of $p_{i}$ concerning bounding box $bb_{k}$ in X–Y by means $lr_{i}=\left(\left(x_{i}-xc_{k}\right)\times \left(cos\left(-\theta_{i}\right)\right)\right)+\left(\left(y_{i}-yc_{k}\right)\times \left(-sin\left(-\theta_{i}\right)\right)\right),lyn_{i}=\left(\left(x_{i}-xc_{k}\right)\times \left(sin\left(-\theta_{i}\right)\right)\right)+\left(\left(y_{i}-yc_{k}\right)\times \left(cos\left(-\theta_{i}\right)\right)\right)$, and then determine if a point belongs, and return the respective index to bounding box by $(□\left(lnx_{i}<\frac{dx_{i}}{2}+0.00001\vee \frac{lny_{i}}{2}+0.00001<dy_{i}\right)\to id=i$. After obtaining the points indexes within the bounding boxes, all inside points are aggregated with PointNet++.

**Point Intrapart Offset**

This consists of both CR and PR to predict point class labels and point bounding box offsets.

**Point Head Simple**

This is only composed of CR. However, it has modifications to its architecture $CR≜\{cr_{1},cr_{2},cr_{3}\}$, where each cr is represented by a tuple (LR,BN,RL), where LR means linear regression, BN means batch normalization and RL means the ReLU method. BN can be defined by (NF), where NB means the number of features, and typically assumes the same value as OT.

**Point Head Box**

This is composed of CR and PR with architecture modifications. $CR\{cr_{1},cr_{2}\}$ where CR≜(LR,BN,RL) where LR means linear regression, BN means batch normalization and RL means the ReLU method. PR is composed of $PR≜\{pr_{1},pr_{2}\}$, where each pr is defined by the same tuple (LR,BN,RL).

#### 4.4.3. RoI Head

The regions of interest (RoI) head is responsible for taking the RoI features of each box proposal of the RPN Head and then optimizing the imperfect bounding box proposals by predicting and fixing the size and location (centre and orientation) residuals relative to the input bounding box predictions. Besides each model’s specificities, any RoI head is composed of a proposal layer that generates/refines a set of RoIs based on RPN RoIs, denoted as PL; an RoI feature extraction method RF; and a head module HM that can be composed but not restricted to the shared fully connected layer SFC, up–down layer UL and DL, class layer CL, regression layer RL, RoI point pool 3D layer (RoIPL), RoI grid pool layer (RoIGL), RoI-aware pool 3D layer (RoiAP3D) and a convolution part (CnvP) and convolution RPN (CnvRPN).

SFC is responsible for feature extraction and can be defined by a set $\left\{sfc_{0},\;\cdots sfc_{f}\right\}$, f=[0,2[, and $sfc_{f}\in SFC$ and $sfc_{f}$ are represented by a tuple (C1D,BN1D,RL,DRO), where C1D means convolution 1D, BN1D means batch normalization 1D, RL means ReLU and DRO means dropout. CL can be defined by the set $\left\{cl_{0},\;\cdots ,\;cl_{c}\right\},c=[0\;,2[$ and each $cl_{c}$ by (C1D,BN1D,RL,DRO). RL produces box predictions and is composed by the set $\left\{rl_{0},\;\cdots ,\;rl_{r}\right\},r=[0,2[$, where each $rl_{r}$ is defined by (C1D,BN1D,RL,DRO). DL and UL mean bottom-up box generation proposal layers from foreground points. A sequence of convolution 2D and ReLU methods can define the DL. A UL is represented as $ul_{1},ul_{2}$ and each ul by the same sequence of convolution 2D and ReLU methods.

RoIPL are specifically pool 3D points and their corresponding point features according to the location of each 3D proposal of PL. Admitting the given output of bounding boxes BB and a specific bounding box $bb_{n}\in BB$, where $BB≜\{bb_{n}=\left(x_{n},\;y_{n},\;z_{n},\;h_{n},\;w_{n},\;l_{n},\;\theta_{n}\right)\}$, where *x*, *y*, *z* are center coordinates of the predicted bounding box, *h*, *w*, and *l* denote the height, width and length of the bounding box, and θ denotes the orientation of the bounding box. Herein, the ROIPL produces an enlarged set of $bbe_{n}\in BBE$ that can be defined by $\left(x_{n},y_{n},z_{n},h_{n}+\eta ,w_{n}+\eta ,l_{n}+\eta ,\theta_{n}\right)$, where η represents a constant value to resize the bounding box. The depth information loss for each bounding box proposal is compensated by including the distance information to the LiDAR sensor to the $uf_{p}\in UF$ that are BEV spatial features. Each $uf_{p}$ is augmented with $d_{b}≜\sqrt{{\left(x_{p}-x_{c}\right)}^{2}+{\left(y_{p}-y_{c}\right)}^{2}+{\left(z_{p}-z_{c}\right)}^{2}}$, $d_{b}\in D$, where $x_{p}$, $y_{p}$, and $z_{p}$ correspond to coordinates of point features of the local encoder module and $x_{c}$, $y_{c}$ and $z_{c}$ are the center coordinates of the LiDAR sensor. Thus, it generates a tensor in the form $\left(VLM_{out},D\right)$ that is fed to PointNet++, as described in Section 4.3.1, to encode the augmented tensor with local features with global semantic BEV features UF. This generates a feature vector for confidence classification and box refinement.

The idea of RoIGL is to aggregate the keypoint features to the RoI grid points with multiple receptive fields. Grid points are uniform sampling, and can be described by $GP≜\{gp_{1},gp_{2},\cdots ,gp_{s}\},s=216$, which means that a grid 6×6×6 is usually adopted. Firstly, the identification of neighboring keypoints to grid $gp_{i}$ in a radius *R* is performed by means $GF≜\{\forall p\leq r,p\in K|R=\left(xr,yr,zr\right)\in R^{3},\;p_{j}=\left(px_{j}\;,py_{j},pz_{j}\right)\in R^{3}\;|\;gp_{s}=\left(gpx_{j}\;,gpy_{j},\;gpz_{j}\right)\in R^{3}\left|\right\|p_{j}-gp_{s}{\|}^{2}\},\;i=[0,\;\mathrm{NK}[$. After all, a PointNet block is used to aggregate the neighboring keypoint set GF in the same way as Equation (2):

(2) $$\begin{array}{c} PTN≜\{ptn_{i}=max\left(ML\left(SG\left(gf_{i}\right)\right)\right)\} \end{array}$$

Then, the two MLP layers, SFC(PTN) and SC(PTN), are performed.

RoIAP3D aims to provide bounding box score confidence and refinement by aggregating the local feature information ($VLM_{out}$) with global semantic BEV features (UF) within the proposals. Two operations are performed within the point features $pf_{i}$ of bounding boxes BB, such that $BB≜\left\{bb_{k}=\left\{pf_{i}\in R^{C}\right\}\right\},i=[0,\;m[,pf_{i}\in PF$ and is scattered to the voxel data structures $VLB≜\{vlb_{k}=\left(x_{j},\;y_{j},\;z_{j}\right),i=[0,m\left[\right\}$ where $x_{j}$, $y_{j}$, $z_{j}$ are encoded in canonical coordinates using the point head module, and *m* is the number of inside points within bounding box $bb_{k}$. The objective is to solve the problem of different proposals generating the same pooled points. For this purpose, average pooling for pooled part features operation—denoted as PPF—and max pooling for pooled RPN features—defined as PRPN—are adopted, and can be described as $PPF≜RoIMax\left(VLB,PF,BB\right),PPF\in R^{S_{x}\times S_{y}\times S_{z}\times C}$ and $PRPN≜RoIAvg\left(VLB,PF,BB\right),PPF\in R^{S_{x}\times S_{y}\times S_{z}\times C}$ where $S_{x}$, $S_{y}$, $S_{z}$ are the resolution of the voxels’ spatial shape. The operations RoIMax and RoIAvg can be described more specifically:

$RoIMax=\left\{\begin{array}{cc} max(\left\{pf_{i}\in vlb_{k}\right\}), & \mathrm{if}\;count(PPF)>0\\ 0, & \mathrm{otherwise} \end{array}\right.$

$RoIAvg=\left\{\begin{array}{cc} \frac{\sum_{i=0}^{count(PPF)}pf_{i}}{count(PPF)},pf_{i}\in vlb_{k}\left(\left\{pf_{i}\in vlb_{k}\right\}\right), & \mathrm{if}\;count(PPF)>0\\ 0, & \mathrm{otherwise} \end{array}\right.$

## 5. Three-Dimensional Object Detection Model Specifications

**Figure 3:**
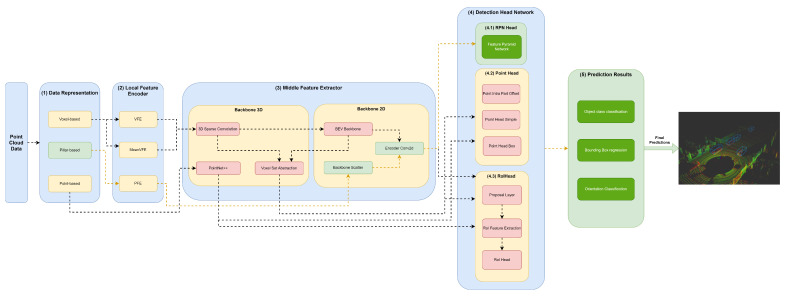
Structure of the PointPillars model represented in the developed framework.

**Figure 4:**
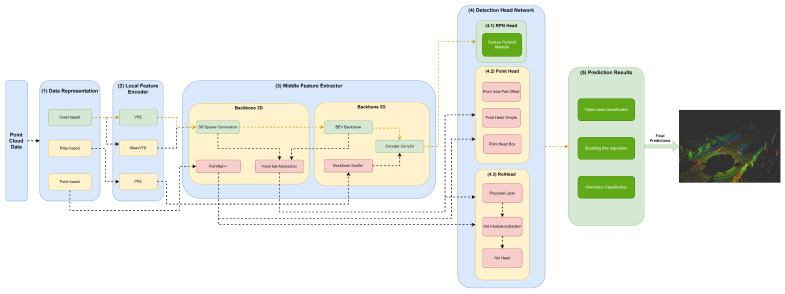
Structure of the SECOND model represented in the developed framework.

**Figure 5:**
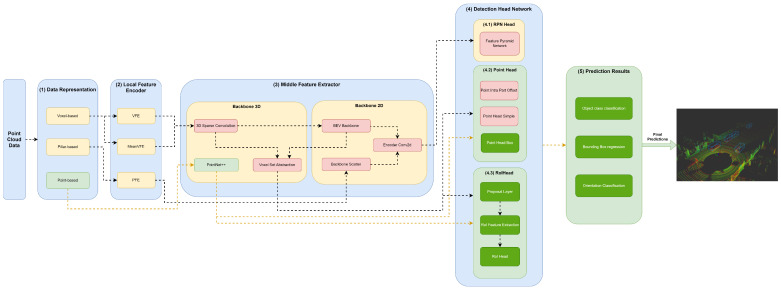
Structure of the PointRCNN model represented in the developed framework.

**Figure 6:**
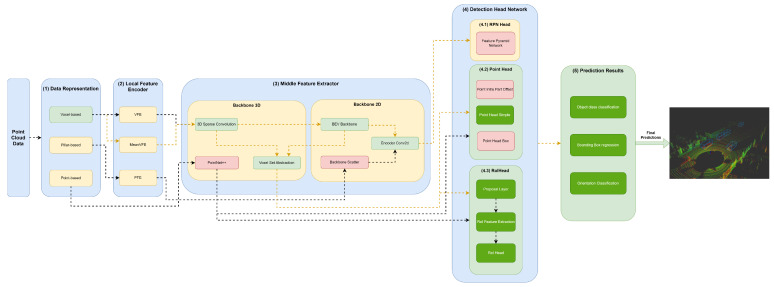
Structure of the PV RCNN model represented in the developed framework.

**Figure 7:**
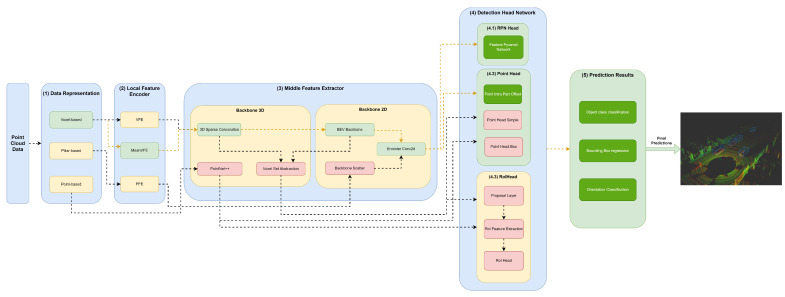
Structure of the ${\mathrm{PartA}}^{2}$ model represented in the developed framework.

**Figure 8:**
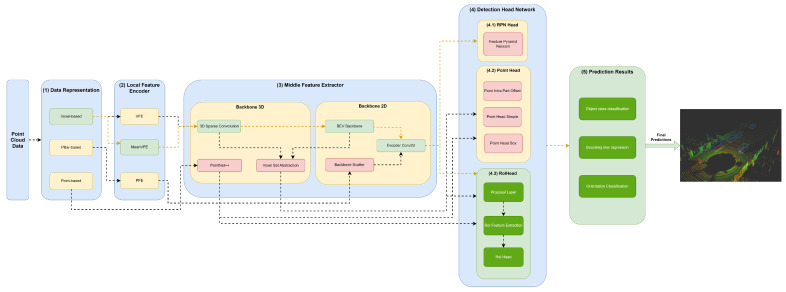
Structure of the VoxelRCNN model represented in the developed framework.

Herein, we will specify each model in the different module frameworks. These models were selected based on the requirements established and defined in Section 1, since they are the models that best guarantee the trade-off between metrics (mAP and inference time). The set of models and their specificities concerning the developed framework are illustrated in Figure 3, Figure 4, Figure 5, Figure 6, Figure 7 and Figure 8. The modules of each model are represented in the figures as green boxes, and the flow of the tensors occurs in the direction of the orange arrows.

### 5.1. Data Representation

Typically, the models of Figure 4 and Figure 6, Figure 7 and Figure 8 are chosen to represent the point cloud in Voxels. In this data structure, the point cloud is delimited (using the cropping technique), and a grid is produced where the data are discretized along the X–Y–Z axis.

Only PointPillars, illustrated in Figure 3, discretizes this delimited space of the point cloud on the X–Y axis, creating a set of pillars.

In the case of the PointRCNN model (Figure 5), it provides the delimited point cloud without any data discretization and structuring process for the middle feature encoder.

### 5.2. Local Feature Encoders

As illustrated in the Figures, three strategies are used by the models to improve the efficiency of the object detectors in the feature extraction of the data structures. Typically, these modules are responsible for the local feature extraction, and then, via concatenation, aggregate these features. Three networks are used: VFE for SECOND (Figure 4), PFE for PointPillars (Figure 3) and mean VFE for PV RCNN (Figure 6), ${\mathrm{PartA}}^{2}$ (Figure 7) and VoxelRCNN (Figure 8).

### 5.3. Middle Feature Extractor

The methods described herein use 3D backbones based on sparse and submanifold convolutions, such as SECOND (Figure 4), PV-RCNN (Figure 6), ${\mathrm{PartA}}^{2}$ (Figure 7) and Voxel-RCNN (Figure 8). PV-RCNN uses the 3D voxel set abstraction backbone to encode the feature maps obtained by the 3D sparse CNN for keypoints. PointRCNN (Figure 5) uses PointNet++ [9] to extract features and pass them to the detection head module.

Only PointPillars (Figure 3) uses 2D backbones, since they require fewer computational resources when compared to 3D backbones. However, they introduce a loss in the information that is easily mitigated, since it is possible to readjust the objects again to the Cartesian 3D system of LiDAR with less loss of information. For this purpose, the resulting PFE features are used by the backbone scatter component, which scatters them back into a 2D pseudoimage. The next detection head component then uses this 2D pseudoimage.

Other models, such as SECOND (Figure 4), PV RCNN (Figure 6), ${\mathrm{PartA}}^{2}$ (Figure 7) and Voxel-RCNN (Figure 8) compress the information in a bird’s-eye view (BEV) using the BEV backbone for feature extraction, then encode and concatenate the features using the encoder Conv2D component. After this process, the resulting features are passed to the detection head.

### 5.4. Detection Head

As mentioned earlier, this module comprises three networks: RPN head, point head and RoI head.

All models except PointRCNN use the RPN head to generate RoIs using a low-level algorithm called selective search [22] to produce proposed regions per frame of the point cloud. Selective search generates subsegments to generate many candidate regions, and following bottom-up grouping, recursively combines similar regions into larger regions to provide more accurate final candidate proposals. Each of these regions is submitted independently to the CNN module. The output feature map is then fed to an SVM classifier to predict the object class within the candidate RoI. Along with object class prediction, the algorithm also predicts four bounding box offset values.

The point head is used to assist the RPN head, as illustrated in Figure 6 and Figure 7, or generate predictions of object classes and predict four values that are the bounding box offsets, as shown in Figure 5 and Figure 8. Point head generates various masks of objects or parts of objects in a multiscale way, followed by a simple bounding box inference to generate proposals, also called point proposals, using each point to contribute to the reconstruction of the 3D geometry of the object.

The RoI head used by the PointRCNN (Figure 5), PV-RCNN (Figure 6), ${\mathrm{PartA}}^{2}$ (Figure 7) and Voxel-RCNN (Figure 8), naturally uses the RoI features of each bounding box proposed in the RPN, and then optimizes the imperfect bounding boxes from previous stages, predicting and correcting the size and location (center and orientation) in relation to the predictions of the input bounding boxes.

## 6. Network Training and Fine Tuning

The models described in this document were trained using the KITTI data sets. In addition, the models were evaluated based on the KITTY benchmarks, namely for detecting 3D objects and BEV, considering a validation set. Regarding the number of epochs used in the training phase, a methodology spread by the literature was considered. Thus, we use 200 epochs, considering the data described in Table 13. Considering training hyperparameters, We define the initial learning rate of 0.01, learning rate decay of 0.1, decay epoch methodology, weight decay of 0.01, gradient clipping normalization with a max value of 10, beta1 of 0.95 and beta2 of 0.85. We use the learning rate decay, weight decay, and gradient clipping normalization as regularization procedures to prevent overfitting. The evaluation metrics in the results were based on the official KITTY evaluation detection metrics. Hence, the metrics used were mAP for a BEV and 3D object detection. The partition of the training data used in this work consisted of a division discussed in [2]. This approach divides the 7481 training examples that are provided into a training set of 3712 samples, with the remaining 3769 samples belonging to the evaluation set. Moreover, the benchmarks presented in this article are based on the evaluation set only.

We select three target classes in all experiments: car, pedestrian and cyclist. Typically, all the models described herein generate two separate networks. One network is optimized for predicting cars and another for pedestrians and cyclists. However, this approach can be improper in self-driving car applications since low-edge devices with few resources must cope with two parallel models. For this reason, we trained all classes in a one-single model for all 3D object detectors.

For the fine-tuning process, we save the results of the mAP for each epoch to understand when models converge. Herein, we provide a study with the consequences of the number of samplings and min points per class sampling compared with the study made in [23]. In [23], we used different class sampling strategies but without changing the number of min points for class sampling.

**Sampling Instance Strategy.** We focus on optimizing the number of sampling instances and min points per class sample. The main objective of the sampling strategy is to soften the KITTI dataset imbalance issue. During training, the point cloud is randomly fed with these instances, which means they are placed into the current point cloud. Although this is true, the min points affect whether or not a certain instance can be used for sampling. If we increase the min number of points in the training process, instances such as pedestrians and cyclists are less sampled because few points exist to describe their shape. On the other hand, if we decrease too many min points, the model suffers in distinguishing between the foreground and the background points. In our experiments, we use the configurations described in Table 9. The min point for class sampling was fixed per class as 5 instead of 10 points for pedestrian and cyclist classes and 5 points for the car.

**Point Cloud Range.** Any object detector’s detection range is impacted by the point cloud range, which reduces it. For all models in our study, the ground truth object locations are represented using the original point cloud range for all frames in the KITTI dataset frame. For instance, it is feasible to confirm using depth data that most ground truth events in automobiles occur between 0 and 70 metres. The number of cases starts to decline sharply beyond 70 metres from the middle of the LiDAR sensor. This can be explained by the fact that beyond this range, relatively few points can accurately characterize an item’s geometry, making object detection challenging. In this experiment, the point cloud range of PointPillars is compared to that of other models whose detection range is unaffected. Table 10 shows the point cloud ranges. We also compare the research in [23] to the quantity of data structures (maximum number of pillars or voxels).

**Data structure sizes.** The object detection model receives the points in $PC_{R}$ and discretizes them in the X–Y axis, thus creating a set of pillars, or discretizes in X–Y–Z and creates a set of voxels. Each data structure DS has a fixed size in $PC_{R}$. The data structure size directly impacts model accuracy and inference time. Increasing the data structure size can result in too much data being encoded and consequently randomly sampled, leading to information loss (the maximum number of points per data structure is set for computational saving purposes). On the other hand, reducing the data structure size can increase the number of non-empty data structures, increasing memory usage and inference time. Two DS configurations were used in our fine-tuning process, as shown in Table 11.

**Number of Data Structures.**

Since most data structures will be empty, a maximum number of data structures is established to investigate the KITTI dataset sparsity problem. In order to generate a dense tensor, using several data structures might cause the majority of them to be filled with zeros, making inference time inefficient. A maximum number of points is also established using the KITTI dataset’s distribution of the number of points per data structure, as shown in Table 12.

## 7. Performance Evaluation, Comparison and Discussion

This section details a series of tests conducted using the random search approach to improve the trade-off between accuracy and inference time performance parameters. The experiments and related network setups and models are shown in Table 13. To comprehend the effects of constructing a model tuned to create three classes of output instead of splitting into two separate networks (one for cars and another for pedestrians and cyclists), PointPillars settings and their outcomes are also supplied.

**Table 13:** The set of experiments conducted and respective network configurations.

Experiment	Model Config.	${\mathrm{PC}}_{R}$ Config.	*SI* Config.	No. Output Classes	$S_{\mathrm{PL}}$ Config.	*P* Config.
1	PointPillars	$PC_{R1}$	$SI_{1}$	3	$S_{DS16}$	$P_{12K}$
2	SECOND	$PC_{R2}$	$SI_{1}$	3	$S_{DS5}$	$P_{16K}$
3	PV-RCNN	$PC_{R2}$	$SI_{1}$	3	$S_{DS5}$	$P_{16K}$
4	PointRCNN	$PC_{R2}$	$SI_{1}$	3	$S_{DS5}$	$P_{16K}$
5	PartA^2^	$PC_{R2}$	$SI_{1}$	3	$S_{DS5}$	$P_{16K}$
6	VoxelRCNN	$PC_{R2}$	$SI_{1}$	3	$S_{DS5}$	$P_{16K}$
7	PointPillars	$PC_{R1}$	$SI_{2}$	3	$S_{DS16}$	$P_{12K}$
8	SECOND	$PC_{R2}$	$SI_{2}$	3	$S_{DS5}$	$P_{16K}$
9	PV-RCNN	$PC_{R2}$	$SI_{2}$	3	$S_{DS5}$	$P_{16K}$
10	PointRCNN	$PC_{R2}$	$SI_{2}$	3	$S_{DS5}$	$P_{16K}$
11	PartA^2^	$PC_{R2}$	$SI_{2}$	3	$S_{DS5}$	$P_{16K}$
12	VoxelRCNN	$PC_{R2}$	$SI_{2}$	3	$S_{DS5}$	$P_{16K}$

The results of the experiments provided in Table 13 are shown in Table 14, Table 15, Table 16 and Table 17. We use the metric AP for three difficulty levels (easy, moderate and hard) and various intersection-over-union (IOU) thresholds according to KITTI benchmarks to provide the results. IOU is 70% for cars and 50% for cyclists and pedestrians. The experiment results from this study are compared to the original ones from the literature in Table 18. The comparison considers the three target classes for both 3D and BEV. The results presented for the conceived experiments consider the overall values per class for the best detection metric.

As demonstrated in the aforementioned results, the model implementations in our framework generally produced better mAP. Regarding the point cloud range in our networks, we reproduced original configurations for all models, with fewer DS when compared with the study in [23] since most DS will be empty. This improvement drastically decreases the inference time when comparing PointPillars with the same research. As shown in Table 19 and Table 20, some models, such as PointPillars, ${\mathrm{PartA}}^{2}$ and PointRCNN, produce very close inference time results. On the other hand, our results for SECOND are better, and are worse in the cases of PV-RCNN and VoxelRCNN. Clearly, there is always a trade-off in terms of inference time for producing three-class inference models. This can be explained by the fact that original models obtained their results by training separated networks, one for cars and another for pedestrians and cyclists (a standard literature practice on KITTI benchmarks). By training three-class models, gradients are affected by all those instances, which leads to our models losing the specialization for prediction. However, as mentioned in [23], producing separate networks is impractical for self-driving applications. One solution can be increasing the model’s layers to improve the capability to learn the required patterns/weights/representations of the data. Although this is true, increasing the model’s depth will decrease the inference speed, which can result in a model not meeting the self-driving requirement for that metric (model’s inference time above 100 ms).

Reducing the minimum points to consider a sample instance brought gains in terms of mAP and for the same model architecture, since more instances can be used for data augmentation. This allows for the expansion of the diversity of the training data and our models to learn more patterns from data.

## 8. Conclusions

The research about deep learning methods for 3D object detection on LiDAR data has increased tremendously in recent years, with many models, repositories and different technologies being developed. Although this benefits scientific development in this area, the various technologies, software, repositories and models are a bottleneck for testing and improving the current methods.

To cope with this limitation, we developed a framework for representing multiple SoA 3D object detectors with highly refactored codes for both one-stage and two-stage methods. The main idea of this framework is to facilitate the implementation, reusing and implementation of new techniques in each framework module with less manual engineering effort. In conclusion, it enables the abstract implementation, reusing and building of any object detector in one single 3D object detector framework.

Nonetheless, it is evident that creating three-class inference models comes with a trade-off regarding inference time. Our study’s results are based on the KITTI validation set, while the original findings were obtained using the KITTI test set. We replicated the original network configurations for all models concerning the point cloud range but with fewer DS than the research mentioned in the previous section. The improvement mentioned earlier leads to a considerable reduction in the inference time when PointPillars is compared to the same research.

The current models for 3D object detection in LiDAR data targeting self-driving applications show their results in powerful servers with dedicated graphics cards and an unlimited power source. However, using this kind of server in the context of a self-driving car is impractical due to limited space and power supply. This shows a limitation regarding deploying 3D object detectors in such an environment. Research must evolve to produce models capable of meeting performance metrics while being deployable in resource-constrained edge devices with limited power supply and computational power.

Besides the capability to easily represent SoA 3D object detectors, other models should be integrated as future work. This requires the constant update of the framework in integrating the new components brought by novel methods since scientific research consistently produces innovation, especially in this area.

## Figures and Tables

**Table 1:** Values used in Bn.

*Bn* Element	Value
ep	0.001
mn	0.01

**Table 2:** Configurations used in SpC for each element.

*SpC* Element	Value
$KsS_{t}$	3
$StS_{r}$	1
$PdS_{v}$	1
$DlS_{l}$	1
$OpS_{a}$	0

**Table 3:** Configurations used in SuM and SpC for each block. N.A.—not applicable.

*SuM* Element	InS	OutS	InM	OutM	Ks	St	Pd	Dl	Op
$blc_{1}\wedge sq1\to SuM$	N.A.	N.A.	4	16	3	1	1	1	0
$blc_{2}\wedge sq1\to SuM$	N.A.	N.A.	16	16	3	1	0	1	0
$blc_{3}\wedge sq1\to SpC$	16	32	N.A.	N.A.	3	2	1	1	0
$blc_{3}\wedge sq2\to SuM$	N.A.	N.A.	32	32	3	1	0	1	0
$blc_{3}\wedge sq3\to SuM$	N.A.	N.A.	32	32	3	1	0	1	0
$blc_{4}\wedge sq1\to SpC$	32	64	N.A.	N.A.	3	2	1	1	0
$blc_{4}\wedge sq2\to SuM$	N.A.	N.A	64	64	3	1	0	1	0
$blc_{4}\wedge sq3\to SuM$	N.the A.	N.A.	64	64	3	1	0	1	0
$blc_{5}\wedge sq1\to SpC$	64	64	N.A.	N.A.	3	2	0	1	0
$blc_{5}\wedge sq2\to SuM$	N.A.	N.A.	64	64	3	1	0	1	0
$blc_{5}\wedge sq3\to SuM$	N.A.	N.A.	64	64	3	1	0	1	0
$blc_{6}\wedge sq1\to SpC$	64	128	N.A.	N.A.	3	2	0	1	0

**Table 4:** Configurations used in NCP for each element.

*NCP* Element	Value
$ncp_{1}$	4096
$ncp_{2}$	1024
$ncp_{3}$	256
$ncp_{4}$	64

**Table 5:** Set of configurations used in OP of a specific SQ of the ML element in a specific PTN.

*NCP* Element	*InC2D*	*OutC2D*
$op_{1}\wedge sq_{1}\wedge ptn_{1}$	4	16
$op_{2}\wedge sq_{1}\wedge ptn_{1}$	16	16
$op_{3}\wedge sq_{1}\wedge ptn_{1}$	16	32
$op_{1}\wedge sq_{2}\wedge ptn_{1}$	4	32
$op_{2}\wedge sq_{2}\wedge ptn_{1}$	32	32
$op_{3}\wedge sq_{2}\wedge ptn_{1}$	32	64
$op_{1}\wedge sq_{1}\wedge ptn_{2}$	99	64
$op_{2}\wedge sq_{1}\wedge ptn_{2}$	64	64
$op_{3}\wedge sq_{1}\wedge ptn_{2}$	64	128
$op_{1}\wedge sq_{2}\wedge ptn_{2}$	99	64
$op_{2}\wedge sq_{2}\wedge ptn_{2}$	64	96
$op_{3}\wedge sq_{2}\wedge ptn_{2}$	96	128
$op_{1}\wedge sq_{1}\wedge ptn_{3}$	259	128
$op_{2}\wedge sq_{1}\wedge ptn_{3}$	128	196
$op_{3}\wedge sq_{1}\wedge ptn_{3}$	196	256
$op_{1}\wedge sq_{2}\wedge ptn_{3}$	259	128
$op_{2}\wedge sq_{2}\wedge ptn_{3}$	128	196
$op_{3}\wedge sq_{2}\wedge ptn_{3}$	196	256
$op_{1}\wedge sq_{1}\wedge ptn_{4}$	515	256
$op_{2}\wedge sq_{1}\wedge ptn_{4}$	256	256
$op_{3}\wedge sq_{1}\wedge ptn_{4}$	256	512
$op_{1}\wedge sq_{2}\wedge ptn_{4}$	515	256
$op_{2}\wedge sq_{2}\wedge ptn_{4}$	256	384
$op_{3}\wedge sq_{2}\wedge ptn_{4}$	384	512

**Table 6:** Set of configurations used in OP of a specific SQ in a specific $FP_{M}$.

*NCP* Element	*InC2D*	*OutC2D*
$op_{1}\wedge sq_{1}\wedge fp_{1}$	257	128
$op_{2}\wedge sq_{2}\wedge fp_{1}$	128	128
$op_{1}\wedge sq_{1}\wedge fp_{2}$	608	256
$op_{2}\wedge sq_{2}\wedge fp_{2}$	256	256
$op_{1}\wedge sq_{1}\wedge fp_{3}$	768	512
$op_{2}\wedge sq_{2}\wedge fp_{3}$	512	512
$op_{1}\wedge sq_{2}\wedge fp_{4}$	1536	512
$op_{2}\wedge sq_{2}\wedge fp_{4}$	512	512

**Table 8:** The different RPN configurations ($c1x_{k}\in C1x$) used. N.A.—not applicable.

Models	$c1x_{1}$	$c1x_{2}$	$c1x_{3}$
PointPillars	(512, 18, 1)	(5, 128, 128, 2)	(5, 128, 128, 2)
SECOND	(512, 18, 1)	(512, 42, 1)	N.A.
PV-RCNN	(512, 18, 1)	(512, 42, 1)	N.A.
PartA^2^	(512, 18, 1)	(512, 42, 1)	N.A.
VoxelRCNN	(5, 128, 256, 2)	(5, 128, 256, 2)	N.A.

**Table 9:** Number of sampling instances (*SI*) per class.

*SI* Configuration	Car	Pedestrian	Cyclist
$SI_{1}$	15	10	10
$SI_{2}$	25	20	20

**Table 10:** The different point cloud ranges ($PC_{R}$) configurations used in fine tuning.

${\mathrm{PC}}_{R}$ Configuration	$X_{\min}$	$X_{\max}$	$Y_{\min}$	$Y_{\max}$	$Z_{\min}$	$Z_{\max}$
$PC_{R1}$	0	69.12	−39.68	39.68	−3	1
$PC_{R2}$	0	70	−40	40	−3	1

**Table 11:** Pillar size ($S_{DS}$) configurations used in fine tuning.

$S_{\mathrm{DS}}$ Configuration	$S_{\mathrm{DS}}\mathrm{length}$	$S_{\mathrm{DS}}\mathrm{height}$	$S_{\mathrm{DS}}\mathrm{depth}$
$S_{DS16}$	0.16	0.16	1
$S_{DS5}$	0.05	0.05	0.1

**Table 12:** Total number of data structures used in fine tuning.

*P* Configuration	Total Number of DS	Max Number of Points per DS
$P_{12K}$	12 K	100
$P_{16K}$	16 K	5

**Table 14:** Results in validation set for BEV detection metric for experiments 1–6.

Model	Epoch	Experiment	Car			Cyclist			Pedestrian			Overall
			Easy	Mod.	Hard	Easy	Mod.	Hard	Easy	Mod.	Hard	
Voxel R-CNN	197	6	96.9	94.89	95.08	73.03	77.68	80.3	85.03	85.54	85.97	87.12
PartA^2^	187	5	97.64	96.72	96.6	81.37	83.02	83.38	90.21	90.81	90.95	90.31
PointPillars	160	1	76.29	79.05	80.80	57.52	58.01	58.10	77.75	72.52	73.62	70.84
PointRCNN	24	4	92.83	88.64	88.55	80.71	79.85	80.9	89.35	89.03	88.67	86.04
PV-RCNN	92	3	94.52	93.91	93.58	78.65	79.46	80.65	80.83	80.32	80.59	84.94
SECOND	154	2	87.97	83.75	84.43	71.29	76.0	78.23	77.99	78.96	79.55	80.74

**Table 15:** Results in validation set for 3D detection metric for experiments 1–6.

Model	Epoch	Experiment	Car			Cyclist			Pedestrian			Overall
			Easy	Mod.	Hard	Easy	Mod.	Hard	Easy	Mod.	Hard	
Voxel R-CNN	140	6	89.55	83.37	82.63	69.72	72.7	73.53	72.16	71.38	72.71	76.29
PartA^2^	182	5	79.15	77.31	77.25	73.6	74.84	76.11	72.63	74.94	76.01	76.46
PointPillars	179	1	63.49	58.98	59.27	52.27	60.16	63.0	41.06	40.38	38.99	53.75
PointRCNN	89	4	84.87	79.86	79.37	68.96	71.11	71.35	76.55	75.01	74.36	75.03
PV-RCNN	139	3	88.86	83.57	82.89	71.52	73.21	74.39	64.34	64.53	64.28	73.86
SECOND	147	2	75.55	72.19	72.43	55.23	62.36	65.06	61.77	62.05	61.34	66.28

**Table 16:** Results in validation set for BEV detection metric for experiments 7–12.

Model	Epoch	Experiment	Car			Cyclist			Pedestrian			Overall
			Easy	Mod.	Hard	Easy	Mod.	Hard	Easy	Mod.	Hard	
Voxel R-CNN	199	12	97.19	96.11	96.32	74.43	77.55	79.92	88.53	88.29	88.42	88.22
PartA^2^	195	11	97.75	96.71	96.61	78.23	80.9	82.74	89.99	90.41	90.76	90.04
PointPillars	21	7	85.76	81.04	82.87	67.04	73.04	75.8	55.39	57.19	58.58	72.42
PointRCNN	16	10	96.3	90.84	90.83	78.31	78.51	79.01	85.88	85.24	85.32	85.05
PV-RCNN	190	9	96.4	93.45	94.08	69.05	72.34	74.74	78.77	80.17	80.7	83.17
SECOND	162	8	90.61	86.51	86.05	78.66	79.76	79.91	66.27	73.66	76.79	80.92

**Table 17:** Results in validation set for 3D detection metric for experiments 7–12.

Model	Epoch	Experiment	Car			Cyclist			Pedestrian			Overall
			Easy	Mod.	Hard	Easy	Mod.	Hard	Easy	Mod.	Hard	
Voxel R-CNN	186	12	83.72	81.21	81.33	68.44	71.01	73.69	67.62	69.28	70.42	75.15
PartA^2^	187	11	83.29	82.53	82.87	74.13	75.38	76.2	69.46	70.95	70.82	76.63
PointPillars	21	7	69.49	66.31	66.94	47.58	52.72	56.98	37.4	36.91	39.48	54.57
PointRCNN	39	10	89.96	83.36	81.59	68.66	71.26	71.32	73.52	74.04	72.66	75.19
PV-RCNN	44	9	83.42	80.46	80.61	63.75	67.41	70.22	63.18	63.38	63.45	71.42
SECOND	162	8	76.02	70.24	72.77	56.1	63.59	65.77	56.2	58.87	58.14	65.56

**Table 18:** Our results in KITTI validation set vs. original results in KITTI test set for 3D and BEV detection metrics.

Model	Our Results (Overall per Class)						Original Results (Overall per Class)					
	3D			BEV			3D			BEV		
	Car	Cyc.	Ped.	Car	Cyc.	Ped.	Car	Cyc.	Ped.	Car	Cyc.	Ped.
Voxel R-CNN	85.18	71.98	72.08	96.54	77.3	88.41	83.19	-	-	89.94	-	-
PartA^2^	82.9	75.24	70.41	96.99	82.59	90.66	79.94	66.54	45.50	88.03	71.34	34.92
PointPillars	67.58	52.43	37.93	83.22	71.96	57.05	75.29	62.56	44.09	86.48	66.07	50.67
PointRCNN	84.97	70.41	73.41	90.01	80.49	89.02	77.77	62.10	41.12	87.41	70.03	47.91
PV-RCNN	85.11	73.04	64.38	94.0	79.59	80.58	82.83	66.65	45.25	90.59	71.26	52.39
SECOND	73.39	60.88	61.72	87.72	79.44	72.24	79.20	62.56	44.09	88.4	68.36	47.63

**Table 19:** Our inference time metric results.

Model	Total (ms) ~	Speed (Hz) ~
PointPillars	17.25	57.97
SECOND	34.1	29.33
PV-RCNN	118.03	8.47
PointRCNN	97.83	10.22
PartA^2^	82.66	12.10
VoxelRCNN	59	16.95

**Table 20:** Original model inference time metric results.

Model	Total (ms) ~	Speed (Hz) ~
PointPillars	16	62.5
SECOND	110	9.09
PV-RCNN	80	12.5
PointRCNN	100	10
PartA^2^	80	12.5
VoxelRCNN	40	25

## Data Availability

Not applicable.
